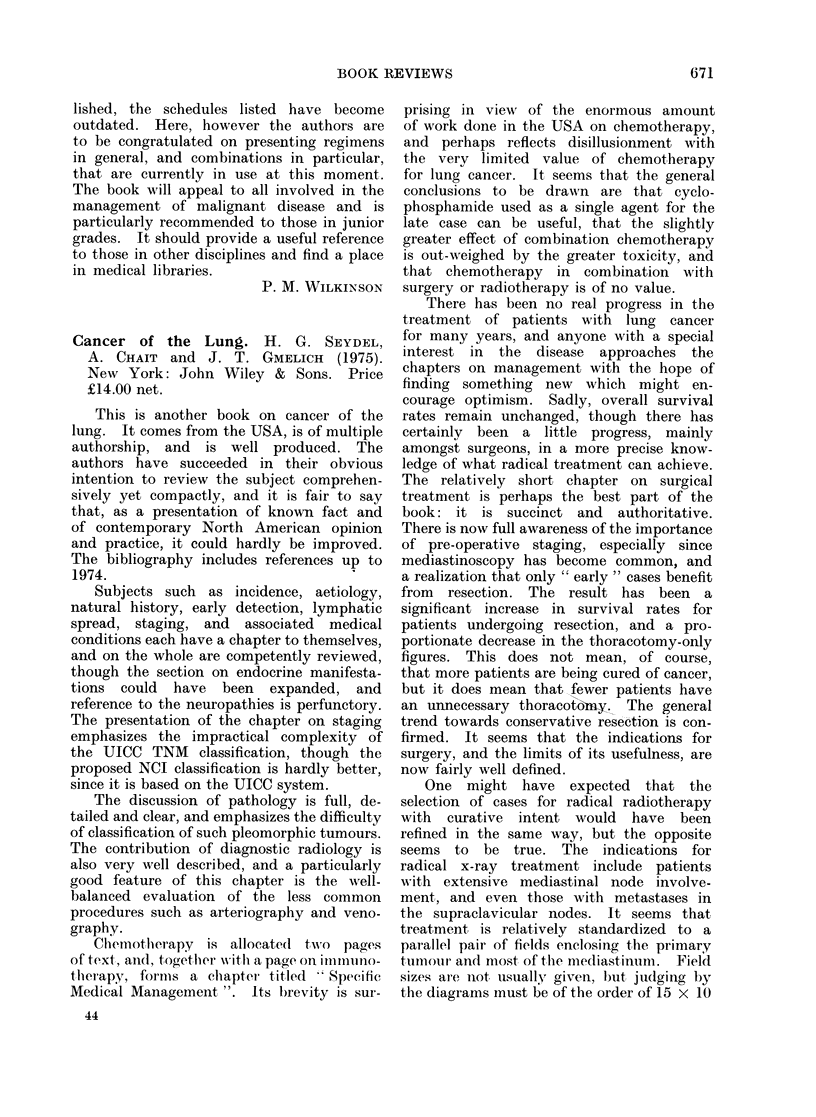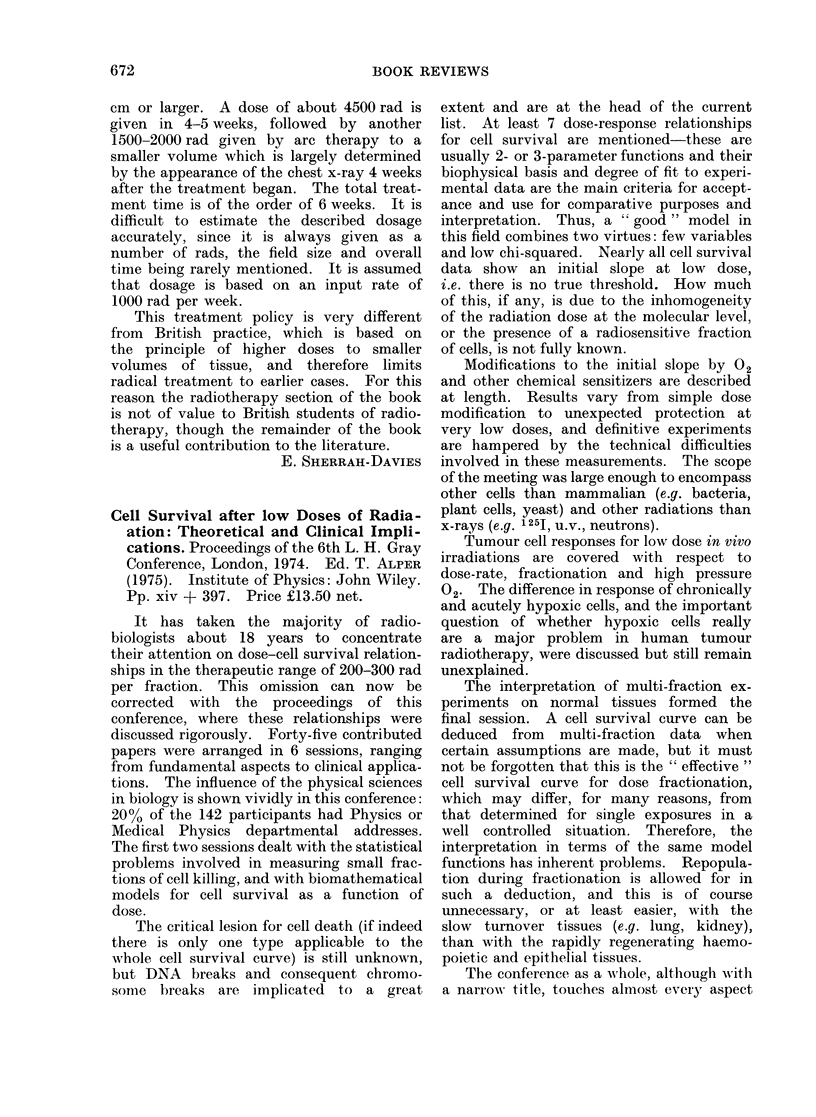# Cancer of the Lung

**Published:** 1976-06

**Authors:** E. Sherrah-Davies


**Cancer of the Lung.** H. G. SEYDEL, A. CHAIT and J. T. GMELICH (1975). New York: John Wiley & Sons. Price £14.00 net.

This is another book on cancer of the lung. It comes from the USA, is of multiple authorship, and is well produced. The authors have succeeded in their obvious intention to review the subject comprehensively yet compactly, and it is fair to say that, as a presentation of known fact and of contemporary North American opinion and practice, it could hardly be improved. The bibliography includes references up to 1974.

Subjects such as incidence, aetiology, natural history, early detection, lymphatic spread, staging, and associated medical conditions each have a chapter to themselves, and on the whole are competently reviewed, though the section on endocrine manifestations could have been expanded, and reference to the neuropathies is perfunctory. The presentation of the chapter on staging emphasizes the impractical complexity of the UICC TNM classification, though the proposed NCI classification is hardly better, since it is based on the UICC system.

The discussion of pathology is full, detailed and clear, and emphasizes the difficulty of classification of such pleomorphic tumours. The contribution of diagnostic radiology is also very well described, and a particularly good feature of this chapter is the well-balanced evaluation of the less common procedures such as arteriography and venography.

Chemotherapy is allocated two pages of text, and, together with a page on immunotherapy, forms a chapter titled " Specific Medical Management ". Its brevity is surprising in view of the enormous amount of work done in the USA on chemotherapy, and perhaps reflects disillusionment with the very limited value of chemotherapy for lung cancer. It seems that the general conclusions to be drawn are that cyclophosphamide used as a single agent for the late case can be useful, that the slightly greater effect of combination chemotherapy is out-weighed by the greater toxicity, and that chemotherapy in combination with surgery or radiotherapy is of no value.

There has been no real progress in the treatment of patients with lung cancer for many years, and anyone with a special interest in the disease approaches the chapters on management with the hope of finding something new which might encourage optimism. Sadly, overall survival rates remain unchanged, though there has certainly been a little progress, mainly amongst surgeons, in a more precise knowledge of what radical treatment can achieve. The relatively short chapter on surgical treatment is perhaps the best part of the book: it is succinct and authoritative. There is now full awareness of the importance of pre-operative staging, especially since mediastinoscopy has become common, and a realization that only " early " cases benefit from resection. The result has been a significant increase in survival rates for patients undergoing resection, and a proportionate decrease in the thoracotomy-only figures. This does not mean, of course, that more patients are being cured of cancer, but it does mean that fewer patients have an unnecessary thoracotomy. The general trend towards conservative resection is confirmed. It seems that the indications for surgery, and the limits of its usefulness, are now fairly well defined.

One might have expected that the selection of cases for radical radiotherapy with curative intent would have been refined in the same way, but the opposite seems to be true. The indications for radical x-ray treatment include patients with extensive mediastinal node involvement, and even those with metastases in the supraclavicular nodes. It seems that treatment is relatively standardized to a parallel pair of fields enclosing the primary tumour and most of the mediastinum. Field sizes are not usually given, but judging by the diagrams must be of the order of 15 × 10

cm or larger. A dose of about 4500 rad is given in 4–5 weeks, followed by another 1500–2000 rad given by arc therapy to a smaller volume which is largely determined by the appearance of the chest x-ray 4 weeks after the treatment began. The total treatment time is of the order of 6 weeks. It is difficult to estimate the described dosage accurately, since it is always given as a number of rads, the field size and overall time being rarely mentioned. It is assumed that dosage is based on an input rate of 1000 rad per week.

This treatment policy is very different from British practice, which is based on the principle of higher doses to smaller volumes of tissue, and therefore limits radical treatment to earlier cases. For this reason the radiotherapy section of the book is not of value to British students of radiotherapy, though the remainder of the book is a useful contribution to the literature.

E. SHERRAH-DAVIES